# Supplementary material for: Flow Virometry Quantification of Host Proteins on the Surface of HIV-1 Pseudovirus Particles
Source: Viruses. 2020 Nov 12;12(11):1296. doi: 10.3390/v12111296 (PMC7697180; doi:10.3390/v12111296)
Supplement: Supplementary file 1 [file viruses-12-01296-s001.pdf]

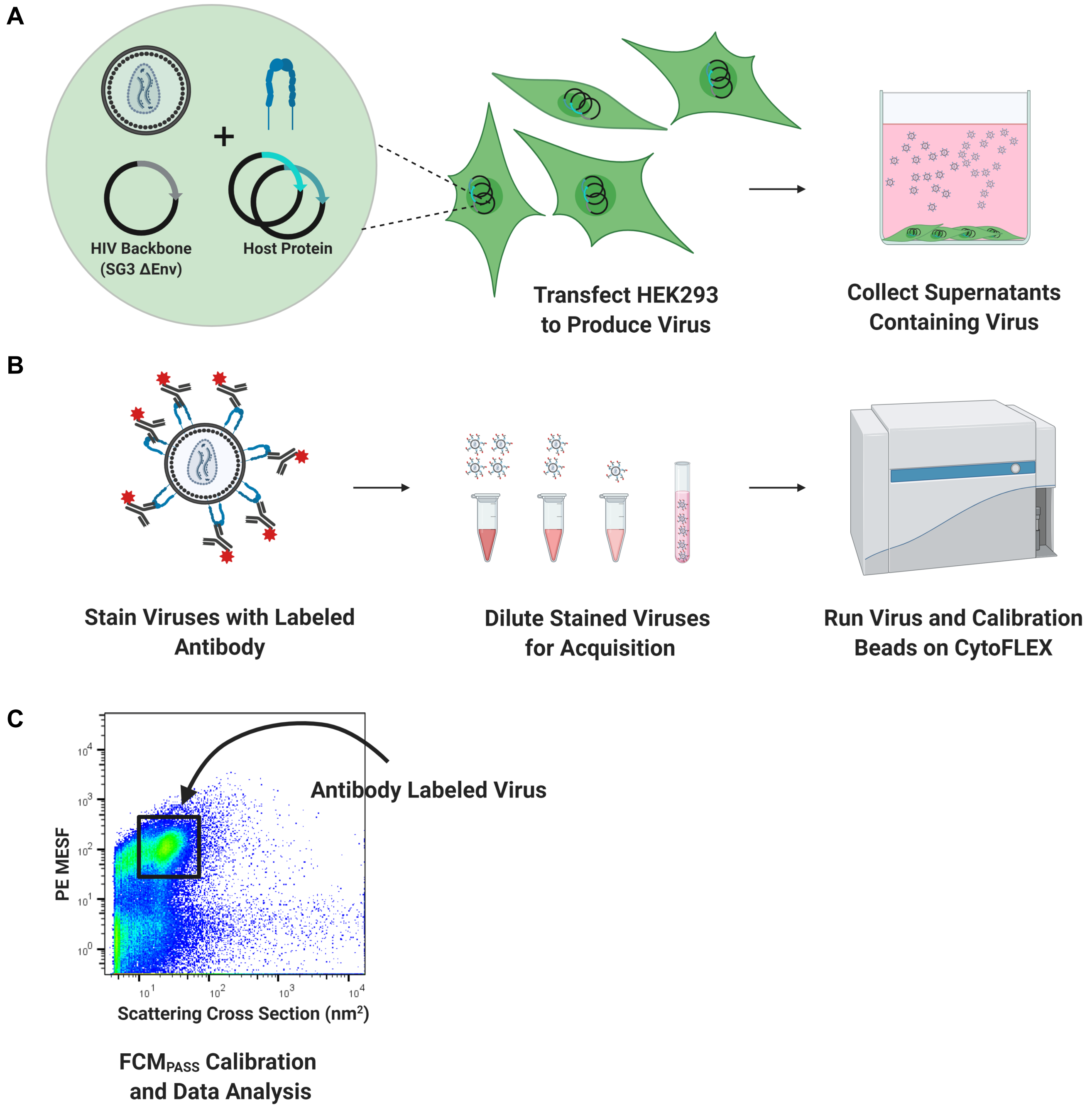

Supplemental Figure 1. Experimental workflow for flow virometry assays. (**A**) Virus particles with or without a cellular protein of interest were generated through transfection of HEK293 cells and the collection of virus-containing culture supernatants. (**B**) All virus stocks were adjusted to 10^9 particles/ml and then stained with monoclonal PE-conjugated antibodies to label host proteins. NIST-traceable sizing beads and fluorescence (Quantibrite and Rainbow) reference beads were used for scatter and fluorescence calibration to allow for standardized reporting of light scatter and fluorescence. Sample and assay controls as outlined in MIFlowCyt-EV framework were also acquired. All samples were acquired on the Beckman Coulter CytoFLEX S flow cytometer at a sampling rate of 10 µl/min. (**C**) Data presentation and analyses were conducted using FlowJo and FCM_{PASS} software.

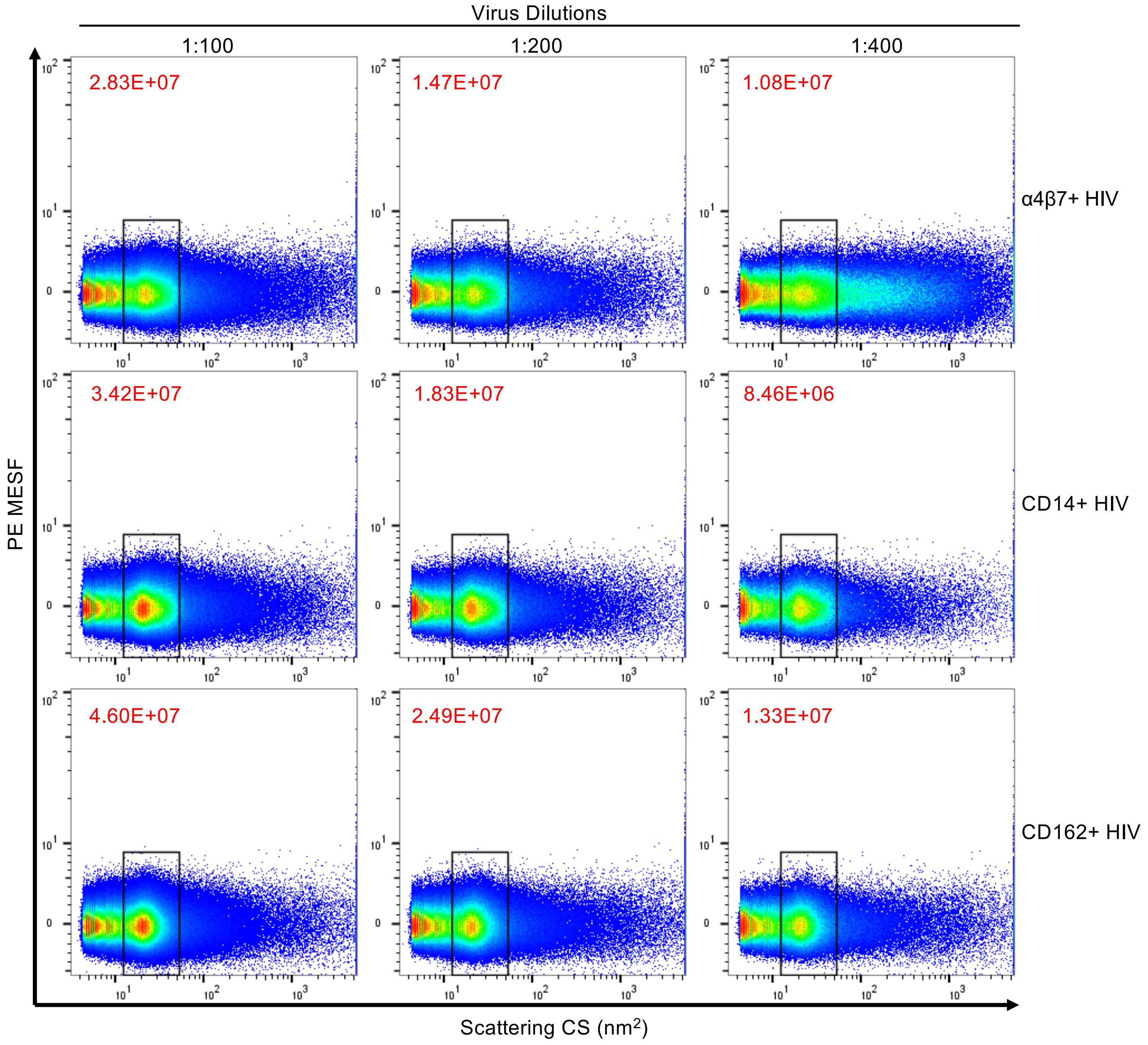

Supplemental Figure 2. Unstained virus dilutions and verification of particle concentrations. Serially diluted (1:100, 1:200, 1:400) preparations of unstained viruses (α4β7+, CD14+, or CD162+ HIV) are shown with gating on the virus population. Particle concentrations of the gated regions are shown in red on each dot plot.

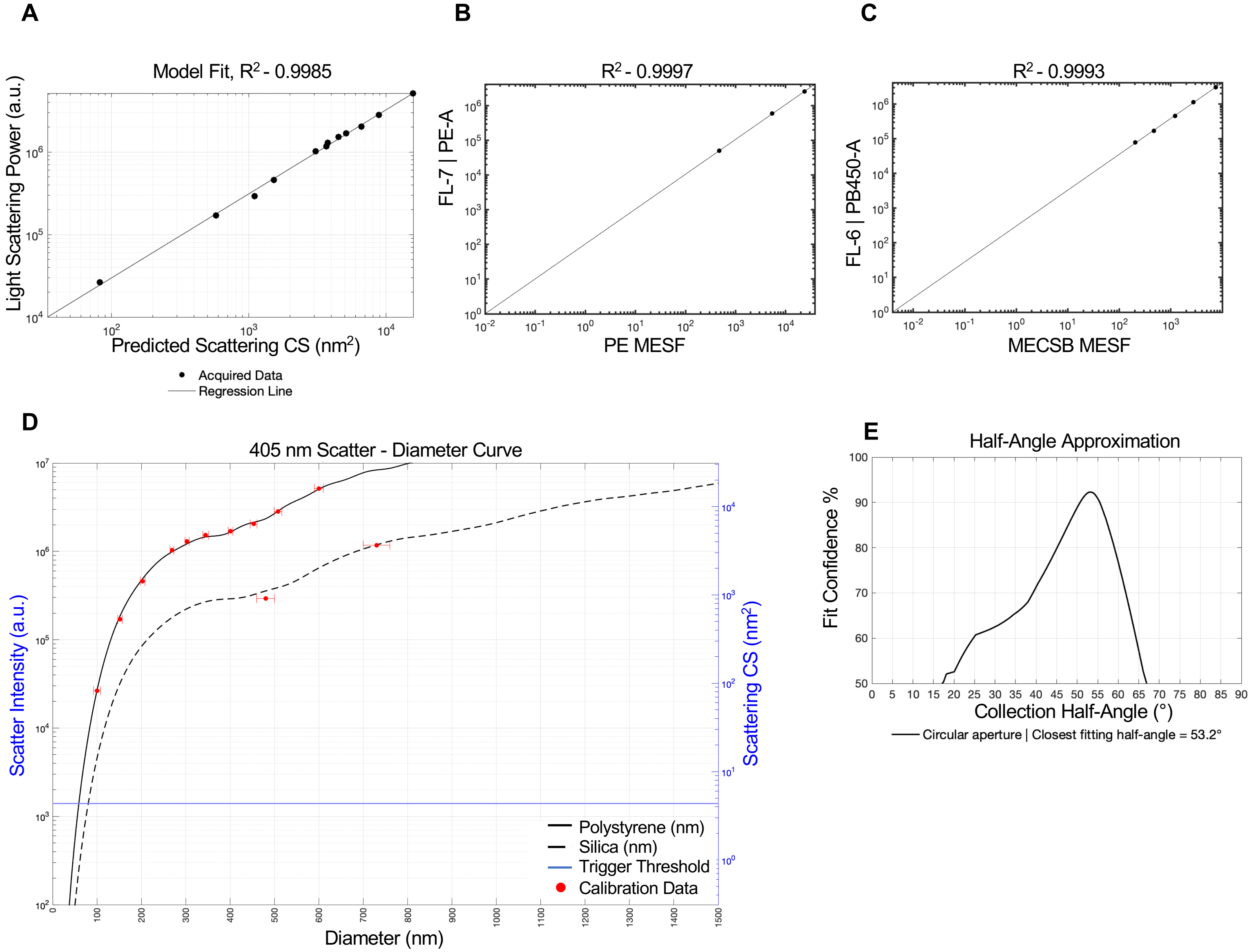

Supplemental Figure 3. Light scatter and fluorescence calibration outputs from FCM_{PASS} software. (**A**) FCM_{PASS} quality control outputs assessing fit of data for scatter modeling through a linear regression of predicted vs acquired light scatter for polystyrene and silica beads of varying diameters. (**B**) Regression of PE Quantibrite beads and (**C**) Rainbow calibration particles generated using FCM_{PASS} to allow for the reporting of fluorescence data in molecules of equivalent soluble fluorophore (MESF). (**D**) The predicted scatter diameter relationship for polystyrene and silica spheres with the instrument's limit of detection for light scatter highlighted in blue is shown. (**E**) Predicted collection half-angle for the CytoFLEX cytometer used for sample acquisition (53.2 degrees).

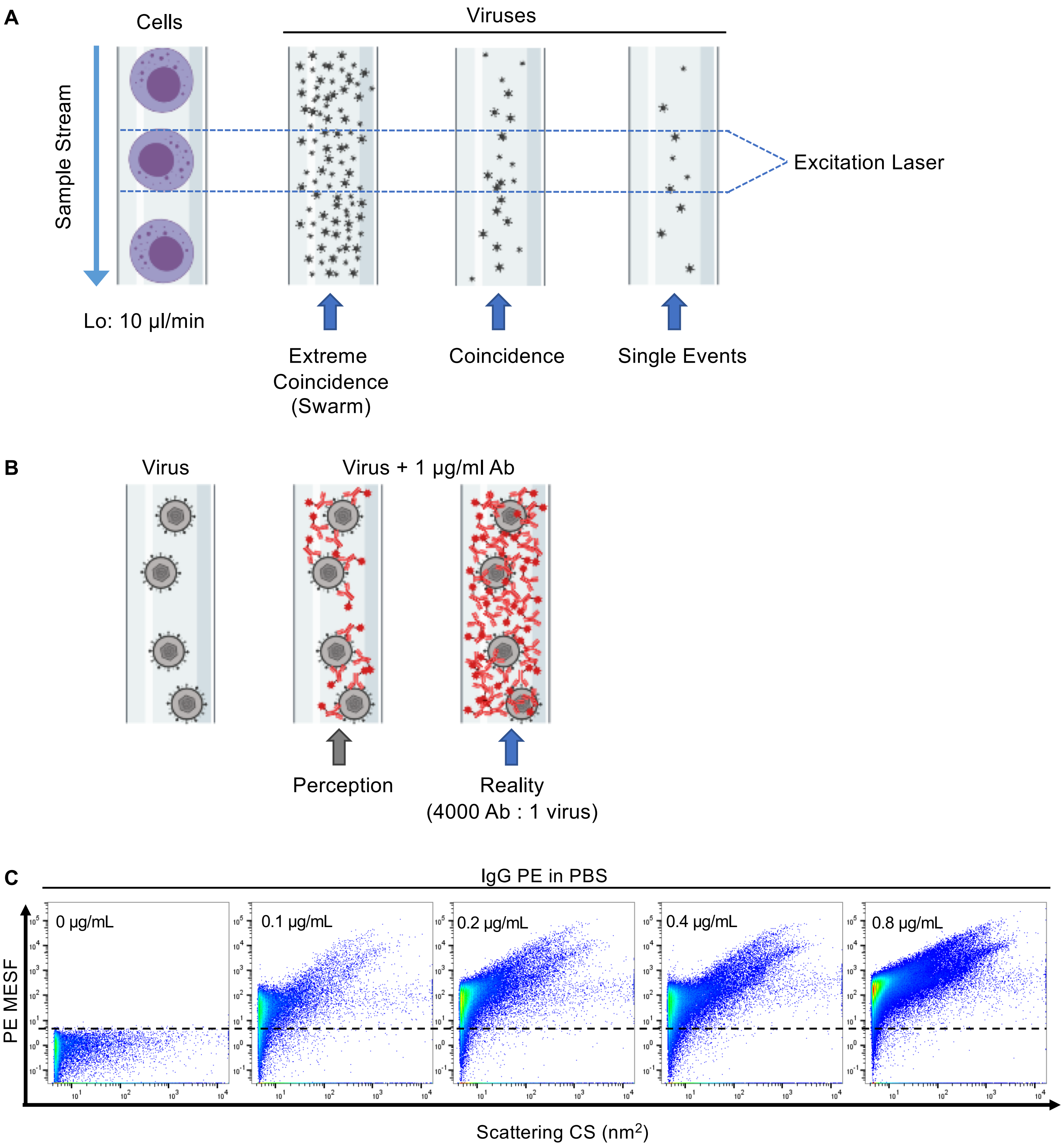

Supplemental Figure 4. Coincidence and antibody swarming in flow virometry. (**A**) Illustration depicting the differences in event detection on a cytometer when running cellular or viral samples. Due to their small size, many virus particles are interrogated by the cytometer at once if samples are not diluted optimally. (**B)** Graphic illustrating the abundance of antibody (Ab) present during viral staining. Since the molecular weight of an IgG antibody is ~150, 000 g/mol, ~4 x10^{12} Ab/ml are present in 1 µg/ml of antibody. Based on this, in a staining application using 10^9 virus particles with 1 µg/ml of antibody, 4000 Abs are present for each single virus. (**C)** Coincidence and swarming demonstrated with a titration of a PE-conjugated IgG antibody alone in PBS. No further dilutions of this stained PBS sample (to reduce background noise) were performed as seen in Figure 3 for virus samples. Data shown are for the anti-α4β7 antibody, representative of the same methods performed for the anti-CD14-PE and anti-CD162-PE antibodies.

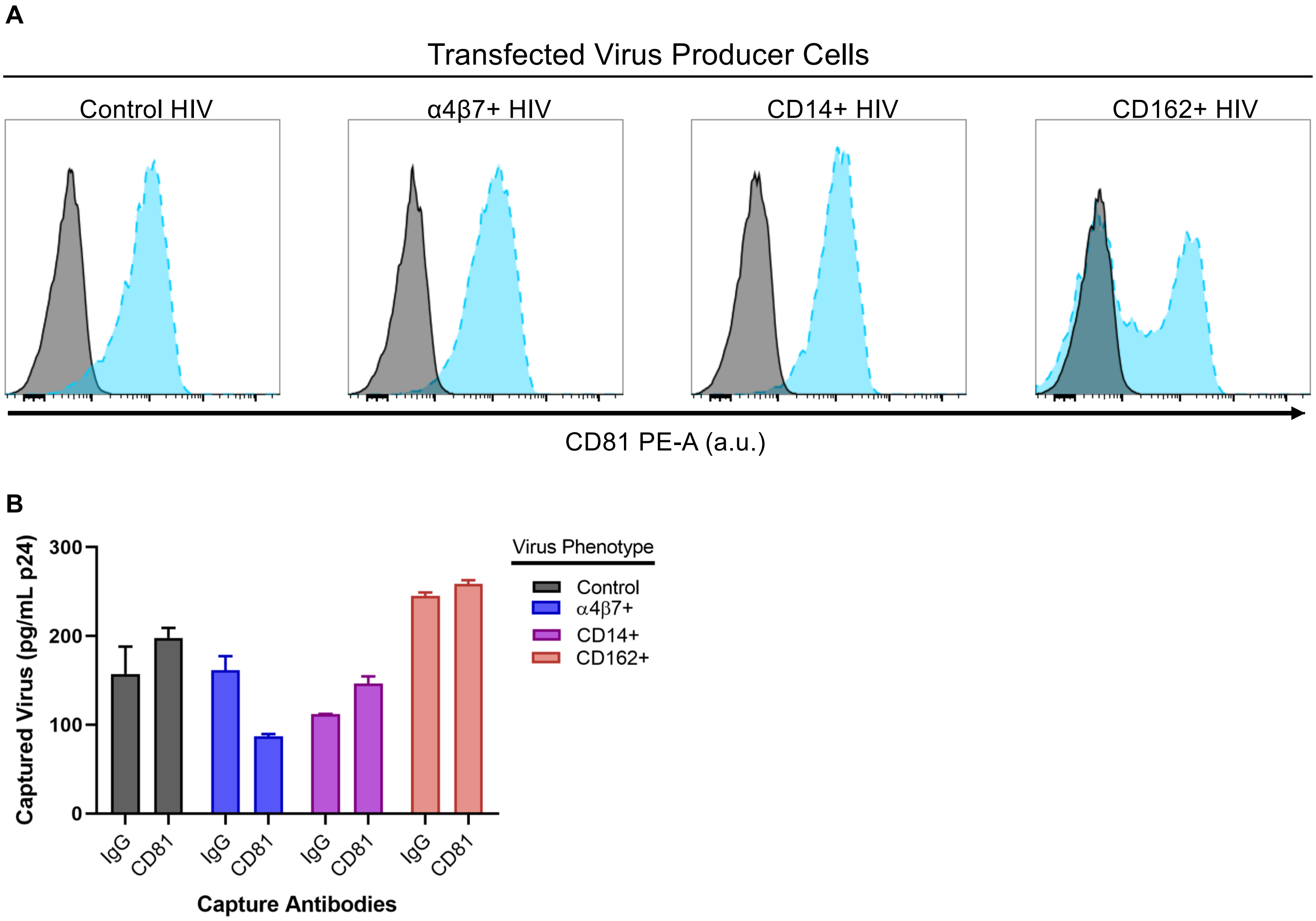

Supplemental Figure 5. CD81 is endogenously expressed on the surface of HEK293 cells and is not detected in virions via antibody capture assays. (**A**) Cell surface expression of the CD81 tetraspanin on virus producer cells (HEK293) co-transfected with the viral construct SG3 $^{\Delta env}$ and the host proteins (α4β7, CD14 or CD162) or transfected with SG3 $^{\Delta env}$ alone (control HIV), as analyzed by flow cytometry. Blue, dotted line histograms indicate positive staining, while control (grey, solid line) histograms indicate negative staining with a non-specific isotype control (mouse IgG). (**B**) Detection of virion-incorporated CD81 by antibody capture assay, with each virus type indicated by a different bar color ('virus phenotypes' containing different host proteins). Control virus contains no host protein, just the SG3$^{\Delta env}$ backbone. Captured virus is presented as the mean amount of p24 (pg/mL) in duplicate samples +/- SD after lysis of bead-associated virus.

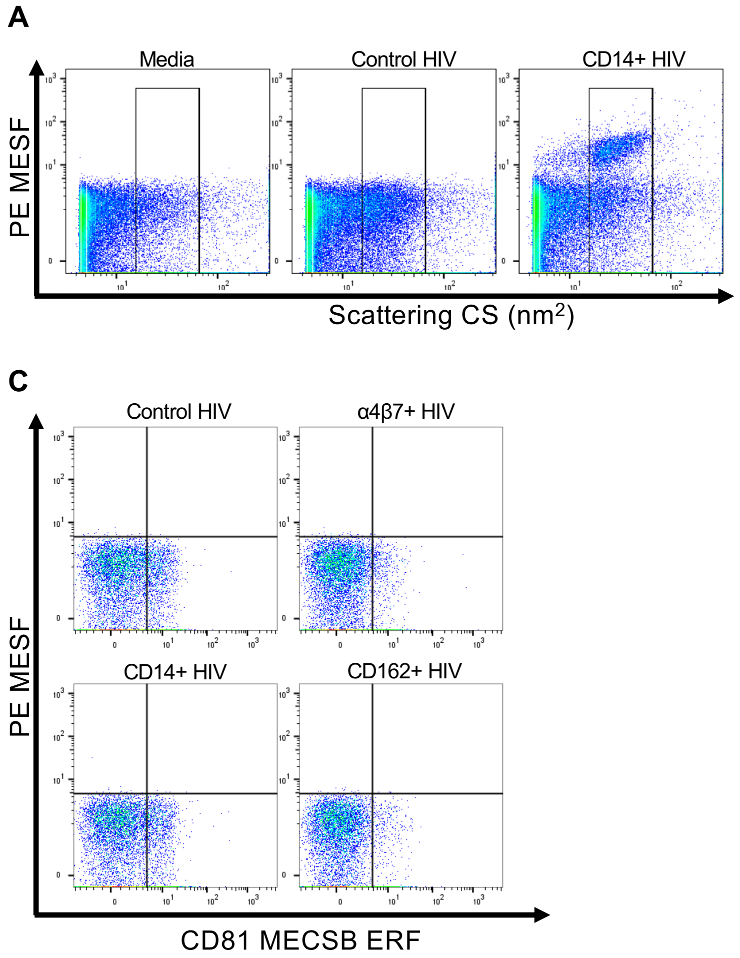

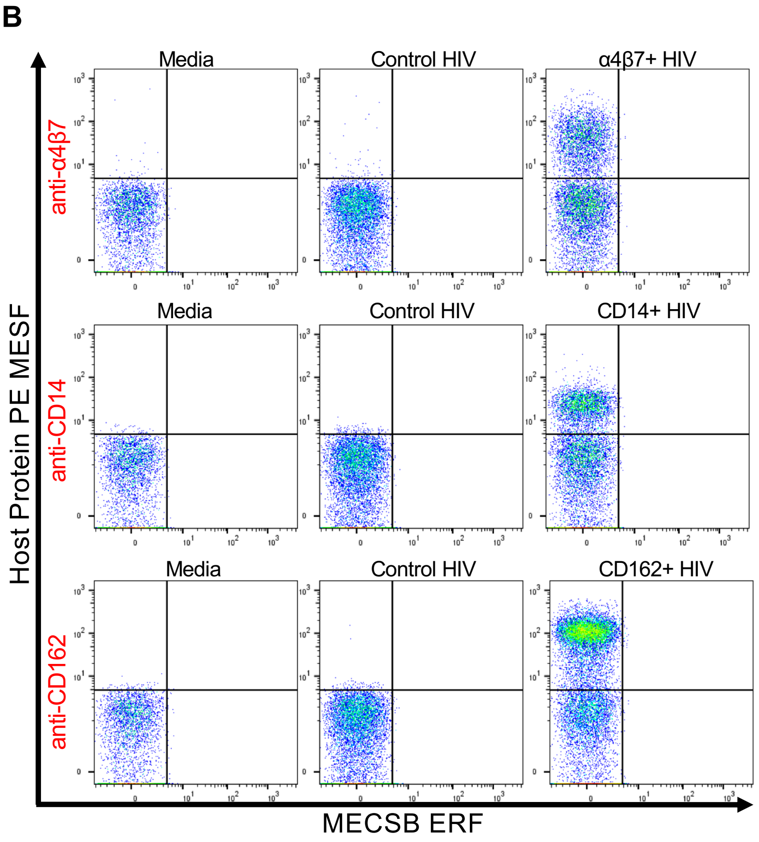

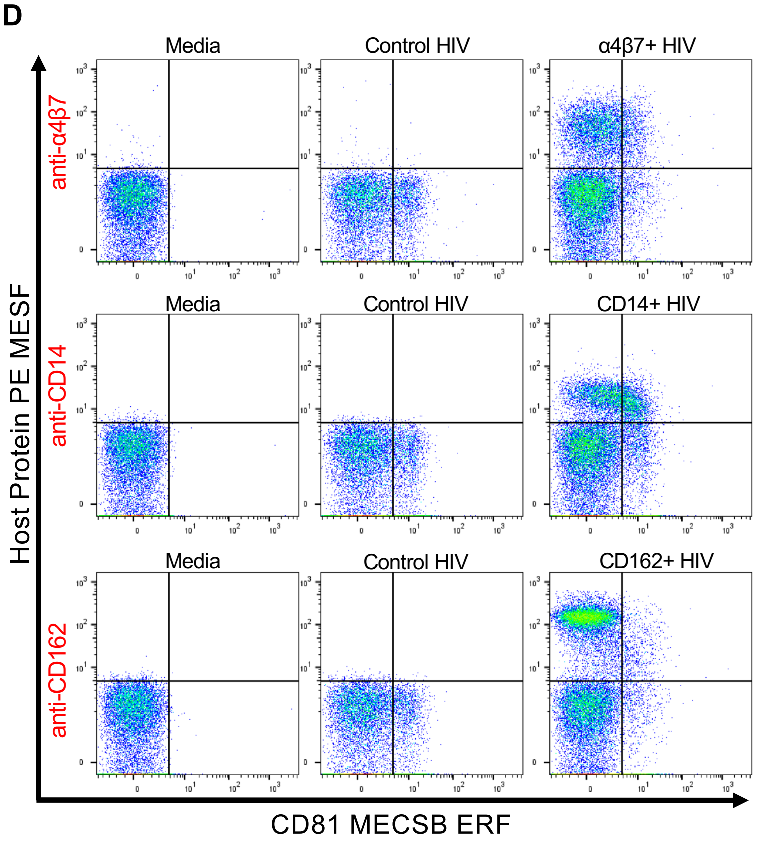

Supplemental Figure 6. Gating strategies used for the phenotypic analysis of cellular proteins on the surface of HIV pseudoviruses. (**A**) Gates for the identified virus population were set based on side-scatter from the control and CD14+ virus plots due to the homogeneity in size of the population. This virus gate was then applied to the media control. Viruses identified within this gate are shown in subsequent analyses of host proteins and the tetraspanin marker CD81. (**B**) Each row shows single staining for a host protein (α4β7, CD14, or CD162) using anti-host protein PE-conjugated antibodies. (**C**) Single staining for CD81 using an anti-CD81 BV421 conjugate on media, control HIV and host protein positive virus samples. (**D**) Double staining using anti-host protein (y axes) and anti-CD81 antibodies (x axes). Control HIV stained with anti-host protein PE was used to set upper and lower PE MESF gates. Cell culture media stained with anti-CD81 BV421 was used to set gates to identify CD81+ events.

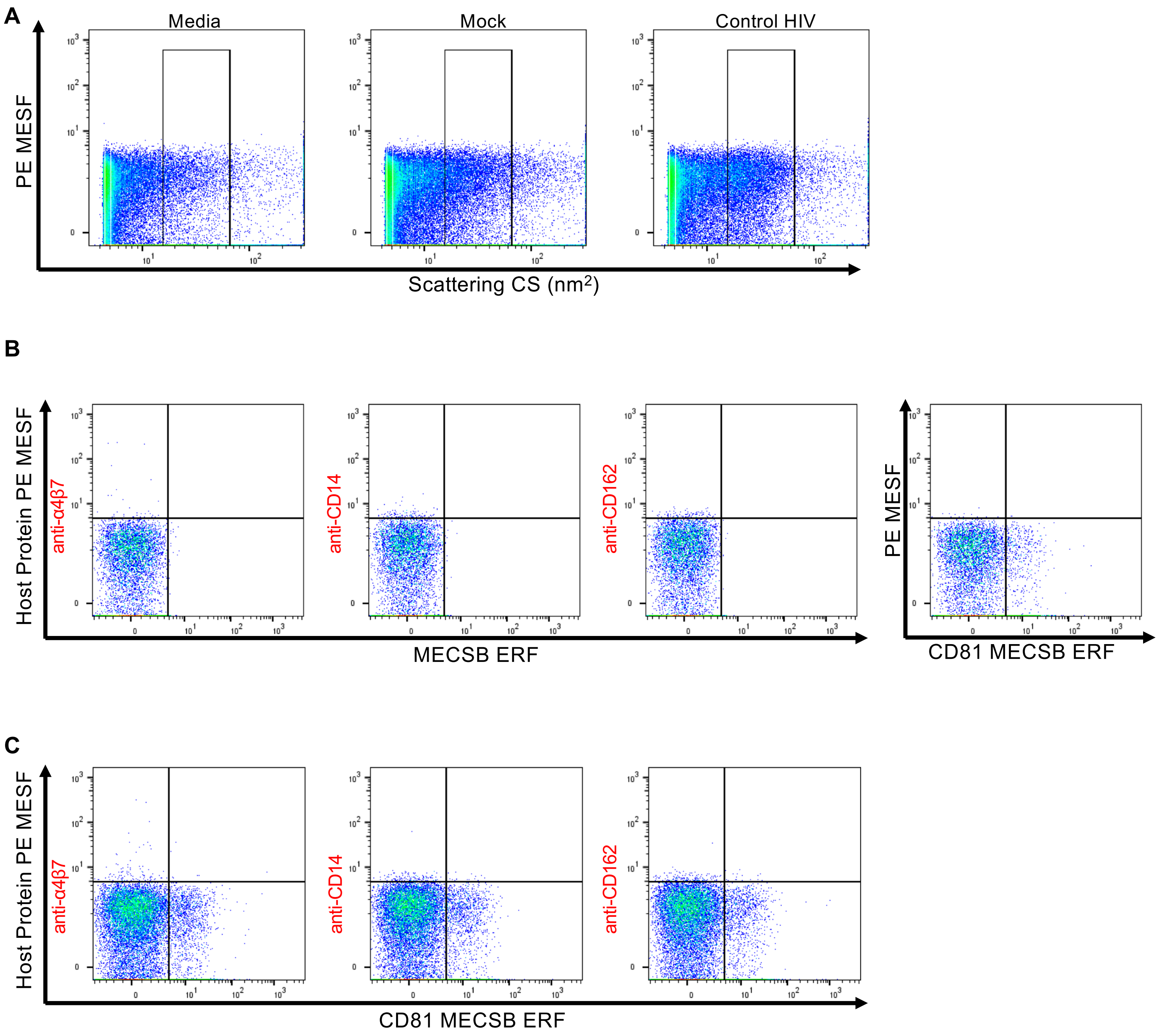

Supplemental Figure 7. Single- and double-staining controls on mock-transfected cell culture supernatants. (**A**) Gating strategy based on side-scatter as shown in Figure S5 was used to identify the EV population present from mock transfection. This gate is also shown on control virus and media alone. The events identified within this gate are then subsequently analysed for expression of specific host proteins and the tetraspanin marker CD81. (**B**) Single-staining of mock-transfected supernatants with anti-host protein (α4β7, CD14, and CD162) PE-conjugated antibodies or an anti-CD81 BV421 conjugate. (**C**) Double-staining of the mock-transfected supernatants shown with both antibody conditions described in (**B**). All plots in B and C are generated from the events in the gate shown in (**A**).

Table S1: FCM_{PASS} Output Report for Light Scatter and Fluorescence Calibration.

FCMPASS software version 3.07

Flow Cytometer Modelling Settings

SSC Parameter	Wavelength	Sheath RI	Determine HA	Aperture Geom	Theta	Phi	Eps	Cali. Factor (nm^2)
FL5-H | VSSC-H	405	1.343092	on	circle	90	90	53.2	0.0031121

Light Scatter Calibration Reference Beads

Diameter (nm)	Diameter CV (%)	Measured RI	Measurement Wavelength	Composition	Acquired Stat	Acquired CV	Acquisition Wavelength	Modelled RI	Manufacturer	Catalogue No.	Lot No.
100	7.8	1.59	589	Polystyrene	26482		405	1.62526527	ThermoFisher Scientific	3100	204935
152	3.3	1.59	589		169894		405	1.62526527		3150	202026
203	2.6	1.59	589		458235		405	1.62526527		3200	205131
269	1.6	1.59	589		1026464		405	1.62526527		3269	202729
303	1.6	1.59	589		1297233		405	1.62526527		3300	204665
345	1.9	1.59	589		1523488		405	1.62526527		3350	224891
401	1.3	1.59	589		1691921		405	1.62526527		3400	203859
453	1.7	1.59	589		2043448		405	1.62526527		3450	204047
508	1.7	1.59	589		2828662		405	1.62526527		3500	204667
600	1.7	1.59	589		5161950		405	1.62526527		3600	205833
480	4.2	1.45	589	Silica	292286		405	1.46110339		8050	203277
730	4.1	1.45	589		1176315		405	1.46110339		8070	207434

Fluorescence Calibration

Parameter	New Parameter Name	Reference Fluor	Ref. Value 1	Ref. Value 2	Ref. Value 3	Ref. Value 4	Ref. Value 5	Ref. Value 6	Ref. Value 7	Acq. Value 1	Acq. Value 2	Acq. Value 3	Acq. Value 4	Acq. Value 5	F/P Ratio	Slope	Interecept	R-Square	Regression Type	Manufacturer	Cat. No.	Lot No.
FL6-A | PB450-A	MECSB MESF	MECSB	205	470	1211	2740	7516	20122	35573	78389	169222	462064	1145123	3108455	1	1.03409123	2.48674589	0.99928937	log	Spherotech	RCP-30-5A	AF01
FL7-A | PE-A	PE MESF	PE	474	5359	23843	62336				50161	605416	2560693			1	1.00600107	2.01461656	0.99973879	log	Becton Dickinson	340495	91367

References

Welsh, J.A., Horak, P., Wilkinson, J.S., Ford, V.J., Jones, J.C., Smith, D., Holloway, J.A. and Englyst, N.A. (2019), FCMPASS Software Aids Extracellular Vesicle Light Scatter Standardization. Cytometry. doi:10.1002/cyto.a.23782

Welsh, J.A., Jones, J.C. and Tang, V.A. (2020), Fluorescence and Light Scatter Calibration Allow Comparisons of Small Particle Data in Standard Units across Different Flow Cytometry Platforms and Detector Settings. Cytometry. doi:10.1002/cyto.a.24029

Welsh J A, Jones J C, Small Particle Fluorescence and Light Scatter Calibration Using FCMPASS Software, Current Protocols in Cytometry, 94, e79. doi: 10.1002/cpcy.79

Sample Acquisition Information

The following extrapolations of thresholds to standard units assume that samples were acquired at the same detector settings as their calibration controls.

Filename	Sample Type	Trigger Parameter	Trigger ID	Trigger Threshold (au)	Detector Setting (au)	Trigger Threshold | Polystyrene (nm)	Trigger Threshold | Silica (nm)	Trigger Threshold | Scattering CS (nm^2)
All files used in this study	Sample	VSSC	FL5-H	1400	195	58.4	79.3	4

Table S2: MIFlowCyt-EV Checklist.

Framework Criteria	What to report	Completed criterion
1.1 Preanalytical variables conforming to MISEV guidelines.	Preanalytical variables relating to EV sample including source, collection, isolation, storage, and any others relevant and available in the performed study.	Pseudovirus containing cell supernatants were generated, collected, and stored as described in section 2.1 of the Materials and Methods section. No further isolation techniques were used.
1.2 Experimental design according to MIFlowCyt guidelines.	EV-FC manuscripts should provide a brief description of the experimental aim, keywords, and variables for the performed FC experiment(s) using MIFlowCyt checklist criteria: 1.1, 1.2, and 1.3, respectively. Template found at www.evflowcytometry.org.	
2.1 Sample staining details	State any steps relating to the staining of samples. Along with the method used for staining, provide relevant reagent descriptions as listed in MIFlowCyt guidelines (Section 2.4 Fluorescence Reagent(s) Descriptions).	Virus samples were stained with PE conjugated antibodies against human cellular proteins (CD14, CD162 and alpha4beta7 integrin) and BV421 conjugated antibody against human tetraspanin CD81. Methods used for optimization of staining protocol are as described in sections 2.3 in the Materials and Methods and 3.3 of the Results section in the manuscript.
2.2 Sample washing details	State any steps relating to the washing of samples.	Virus samples were not washed.
2.3 Sample dilution details	All methods and steps relating to sample dilution.	Unlabelled virus supernatants were serially diluted prior to acquisition on the flow cytometer to estimate virus particle concentration. See section 3.1 of the manuscript for detailed description and Figure S2. Antibody labeled virus samples were diluted with PBS prior to acquisition on the flow cytometer. See section 2.3 and 3.3 of the manuscript for detailed description and Figure 3.
3.1 Buffer alone controls.	State whether a buffer-only control was analyzed at the same settings and during the same experiment as the samples of interest. If utilized it is recommended that all samples be recorded for a consistent set period of time e.g. 5 minutes, rather than stopping analysis at a set recorded event count e.g. 100,000 events. This allows comparisons of total particle counts between controls and samples.	Dilution buffer only (PBS), as well as a media only sample control were run. Media contained 5% FBS, which was EV-depleted by ultracentrifugation (73,000 x g for 24 hours).
3.2 Buffer with reagent controls.	State whether a buffer with reagent control was analyzed at the same settings, same concentrations, and during the same experiment as the samples of interest. If used state what the results were.	Media containing antibody controls were run for each individual antibody used, as well as in combination when dual labeling was performed.
3.3 Unstained controls.	State whether unstained control samples were analyzed at the same settings and during the same experiment as stained samples. If used, state what the results were, preferably in standard units.	Unstained virus samples were analysed using the same settings as stained samples. Refer to Figures 1, 3, and 4 for results showing unstained controls in standard units of MESF, ERF, and scattering cross section.
3.4 Isotype controls.	The use of isotype controls is applicable to immunofluorescence labelling only. State whether isotype controls were analyzed at the same settings and during the same experiment as stained samples. If utilized, state which antibody they are matched to, the concentration used, and what the results were (Section 4.2, 4.3, 4.4). Due to conjugation differences between manufacturers if should be stated if the isotype controls are from the same manufacturer as the matched antibodies.	Isotype controls were not used. Instead, negative controls virus, not expressing cellular protein targets were used.
3.5 Single-stained controls.	State whether single-stained controls were included. If used state whether the single-stained controls were recorded using the same settings, dilutions, and during the same experiment as stained samples and state what the results were, preferably in standard units (Section 4.2, 4.3, 4.4).	Single stained controls were included, recorded in the same settings, and dilutions. See Figures 1, 3, 4, S5 and S6 for results showing single stained controls in standard units of MESF and ERF.

3.6 Procedural controls.	State whether procedural controls were included. If used, state the procedure and if the procedural controls were acquired at the same settings and during the same experiment as stained samples.	Procedural controls of negative control virus and mock transfection controls containing no virus (EVs alone) were included, recorded with the same settings, and dilutions. See Figures 3, 4, S5 and S6 for results showing procedural Procedural controls in standard units of MESF and ERF.
3.7 Serial dilutions.	State whether serial dilutions were performed on samples and note the dilution range and manner of testing. The fluorescence and/or scatter signal intensity would ideally be reported in standard units (see Section 4.3, 4.4) but arbitrary units can also be used. This data is best reported by plotting the recorded number events/concentration over a set period of time at different sample dilution. The median fluorescence intensity at each of the dilutions should also ideally be plotted on the same or a separate plot.	Serial dilutions were included, recorded er the same settings, and dilutions. See Figures 1, 3, 4, S7 and S6 for results showing serial Serial dilutions er standard units of MESF and ERF.
3.8. Detergent treated EV-samples	State whether samples were detergent treated to assess lability. If utilized, state what detergent was used, the end concentration of the detergent, and what the results were of the lysis.	NA - these were not EV samples
4.1 Trigger Channel(s) and Threshold(s).	The trigger channel(s) and threshold(s) used for event detection. Preferably, the fluorescence calibration (Section 4.3) and/or scatter calibration (Section 4.4) should be used in order to report the trigger channel(s) and threshold(s) in standardized units.	Trigger Channel(s) and Threshold(s) in calibrated and arbitrary units are summarized for each sample in the table on the 'Sample Acq Sheet'.
4.2 Flow Rate / Volumetric quantification.	State if the flow rate was quantified/validated and if so, report the result and how they were obtained.	Flow Rate Volumetric quantification was performed using the application in the Beckman Coulter CytExperts acquisition software.
4.3 Fluorescence Calibration.	State whether fluorescence calibration was implemented, and if so, report the materials and methods used, catalogue numbers, lot numbers, and supplied reference units for the standards. Fluorescence parameters may be reported in standardized units of MESF, ERF, or ABC beads. The type of regression used, and the resulting scatter plot of arbitrary data vs standard data for the reference particles should be supplied.	Fluorescence calibration was performed on 2 parameter(s) using FCMPASS software as previously demonstrated [1,2]. Details on the calibration reagents and regression can be found in the summary table on the 'Fl Cali' sheet and in FCMPASS supplementary figure 2.
4.4 Light Scatter Calibration.	State whether and how light scatter calibration was implemented. Light scatter parameters may be reported in standardized units of nm2, along with information required to reproduce the model.	Light scatter calibration was performed using FCMPASS software as previously demonstrated [1,2]. Details on the reagents and modelling parameters can be found in the summary table on the 'SSC Cali' sheet and in FCMPASS supplementary figure 1.
5.1 EV diameter/surface area/volume approximation.	State whether and how EV diameter, surface area, and/or volume has been calculated using FC measurements.	Diameter approximation was not performed as FCMpass did not have the RI values for virus. Arbitrary units of light scatter were reported as scattering cross section (nm^2)
5.2 EV refractive index approximation.	State whether the EV refractive index has been approximated and how this was done.	NA- these are not Evs, refractive index approximation was not performed.
5.3 EV epitope number approximation.	State whether EV epitope number has been approximated, and if so, how it was approximated.	Epitope approximation was not specifically addressed, however fluorescence data was reported in PE MESF which has a approximated equivalence of 1 epitope to 1 PE MESF.
6.1 Completion of MIFlowCyt checklist.	Complete MIFlowCyt checklist criteria 1 to 4 using the MIFlowCyt guidelines. Template found at www.evflowcytometry.org.	Completion of MIFlowCyt checklist were included.

6.2 Calibrated channel detection range	If fluorescence or scatter calibration has been carried out, authors should state whether the upper and lower limits of a calibrated detection channel were calculated in standardized units. This can be done by converting the arbitrary unit scale to a calibrated scaled, as discussed in Section 4.3 and 4.4, and providing the highest unit on this scale and the lowest detectable unit above the unstained population. The lowest unit at which a population is deemed 'positive' can be determined a variety of ways, including reporting the 99th percentile measurement unit of the unstained population for fluorescence. The chosen method for determining at what unit an event was deemed positive should be clearly outlined.	Refer to data presented in Figures 1, 3, 4, S5 and S6 for calibrated detection channel ranges.
6.3 EV number/concentration.	State whether EV number/concentration has been reported. If calculated, it is preferable to report EV number/concentration in a standardized manner, stating the number/concentration between a set detection range.	Virus particle concentrations in serial dilutions of virus containing supernatants were reported for the gated regions denoted (identified by SSC) in Figures 1D and S2.
6.4 EV brightness.	When applicable, state the method by which the brightness of EVs is reported in standardized units of scatter and/or fluorescence.	Brightness as reported in MESF and ERF for fluorescence antibody labeling of cellular proteins was identified in section 3.4, (Fig 3, 4, S5, S6), and scattering cross section for light scatter (Fig 1, 3, 4, S2, S5, S6).
7.1. Sharing of data to a public repository.	Provide a link to the experimental data in a public data repository.	Data has been shared on flowrepository.org (FR-FCM-Z32D)
